# Relation between Shyness and Music Academic Engagement: The Mediation of Achievement Goals—A Cross-Sectional Survey Study

**DOI:** 10.3390/ijerph20010824

**Published:** 2023-01-01

**Authors:** Yan Guo, Yuehan Zhao, Xiantong Yang

**Affiliations:** 1 Department of Music, Taiyuan Normal University, Jinzhong 030619, China; 2 Faculty of Education, University of Cambridge, Trinity Lane, Cambridge CB2 1AG, UK; 3 Faculty of Psychology, Beijing Normal University, Beijing 100875, China

**Keywords:** shyness, music, academic engagement, achievement goals, college students

## Abstract

Music discipline that emphasizes expression, performance and collaboration may cause difficulties for shy students who are prone to anxiety about social interaction, which might cause low music academic engagement and achievement. According to Models of Personality and Affect regarding the role of psychological constructs in educational contexts, shyness and academic engagement are the first and third-level variables, respectively. We hypothesized that achievement goals might be the second-level variable between shyness and academic engagement. Two hypotheses were proposed in the study: (1) shyness is negatively related to music academic engagement; (2) the music achievement goals mediate shyness and music academic engagement. The research was conducted in May 2022. A total of 515 college students who major in music were randomly recruited from a public university in Shanxi province, China. A 20 min self-report questionnaire was conducted as the data collection method. The research results revealed the following: (1) shyness was negatively associated with musical academic engagement; (2) the music mastery goals and the music performance avoidance goals (excluding the performance approach goal) partially mediated the association between shyness and music academic engagement in music learning. These findings have implications for the research and practice of music academic engagement of shyness.

## 1. Introduction

Shyness as a personality trait is defined as constraint and anxiety when confronted with new objects or perceived social evaluations in social situations and is mainly reflected in the conflict between approach and avoidance that arises for individuals in such situations [1]. Shy individuals desire social contact but are also anxious or fearful of approaching others and are prone to show fear, anxiety, tension and social withdrawal in social situations [2,3,4]. School as a social setting for students to learn and live can be stressful for shy students [5]. Hughes and Coplan found that shy students experience social withdrawal in the school environment, which in turn leads to lower academic engagement [6,7].

Fredricks and Blumenfeld defined Academic engagement as an interaction between students and the educational environment [8]. Academic engagement in music educational environment emphasizes collaboration, performance, and responsiveness and is highly demanding of social interaction [9]. Meanwhile, according to the academic engagement positively predict academic achievement [10], shy students’ music academic engagement may influence their music academic achievement. Therefore, due to the characteristics of music as an expressive discipline, more engagement [11,12] and less shyness [13] is required for college students majoring in music when they performing on the stage. As shyness may cause social avoidance, thereby negatively predicting students’ music academic engagement [6], students with less shyness trait would engage deeper and perform better [12]. For music education, college students who major in music are involved in a greater concentration of music education activities compared with other disciplines students. So, many more concerns enhancing music engagement and alleviating shyness should be focused on college students majoring in music. Thus, it is important for music college students with the personality of shyness to explore the association between shyness and music academic engagement, as it may predict their music academic engagement and music academic achievement. Matthews, Zeidner and Roberts proposed a Models of Personality and Affect regarding the role of psychological constructs in educational contexts [14], namely, first-level variables—dispositional constructs (e.g., personality traits), second-level variables—motivational processes, and third-level variables—educational outcomes (e.g., classroom behavior, etc.), while with the help of the second-level variables, the first-level variables have an impact on third-level variables. Shyness as a personality trait belongs to the first-level variable [1]. At the same time shyness has been shown to be associated with academic engagement [6] and might also be associated with musical academic engagement. Academic engagement as a part of the educational outcomes is the third-level variable. According to previous studies, the academic behavior of students with shy personalities is associated with achievement goals [15]. Meanwhile, achievement goals could predict students’ academic engagement [16,17]. Following this line of thought, in the context of music learning, whether achievement goals could be the second-level variables and mediate the shyness and academic engagement attract the authors’ attention. By exploring mechanisms that mediate shyness and music academic engagement, teachers could then provide corresponding support for shy students, thereby improving the potentially low music academic engagement, and promoting their music academic achievement. However, in the context of music learning, there is limited research focus on shyness and music academic engagement. In addition, the integrated model connected the above variable relation in the participants of college students majoring in music is lacking. Therefore, based on Models of Personality and Affect, the present study aimed to verify the association between shyness and music academic engagement and explore whether achievement goals mediate shyness and music academic engagement.

## 2. Literature Review

### 2.1. Shyness and Music Academic Engagement

Academic engagement is defined as the interactive relationship between students and the educational environment and the state of student motivation for academic activities [8]. It is both a shapeable state in the classroom environment and an important predictor of student academic progress and achievement [18,19]. Academic engagement is a multidimensional structure with three main components [8], including (1) Cognitive engagement (intellectual effort to acquire the task); (2) Behavioral engagement (behavior in the classroom and participation in the school community, which can be further segmented into effort, attention, persistence, and peer cooperation, etc. (3), and Affective engagement (perceptions and attitudes towards the learning environment) [20]. Research has found that academic engagement is not only positively associated with academic achievement [10,21], but also reduce college dropout rates [22]. In addition, research on academic engagement helps to understand the quality of students’ learning experiences and helps teachers to determine and provide instructional resources and course content [23] (Coates, 2007). Thus, it is necessary to exploring the factors that influence the students’ academic engagement [24]. Personality is a predictor of academic engagement that impacts how individuals interpret their environment and find ways to self-regulate or adapt to different needs [25]. Shyness, as one of the personality traits, is considered to have a significant negative association with academic engagement [6]. The following discussion attempts to unpack the reasons for it.

Shyness is defined as constraint and anxiety when confronted with new objects or perceived social evaluations in social situations and is mainly reflected in the conflict between approach and avoidance that arises for individuals in such situations [1]. Shy individuals desire social contact but are also anxious or fearful of approaching others [2] and are prone to show fear, anxiety and tension in social situations [3]. School, as the social context in which students learn and live, is considered to be stressful for shy students [5]. Shyness in the school environment leads to social withdrawal, which is directly related to lower academic engagement and academic achievement [6]. In addition, peer relationships and interactions, such as peer acceptance and peer rejection, have an impact on the academic performance and academic engagement of shy students [26]. Furthermore, lack of positive peer relationships or negative peer experiences can also cause emotional stress or anxiety for shy students, which can negatively affect their participation in learning activities, for example, when they are involved in academic activities such as discussions or group work [27,28]. In addition to social withdrawal and peer interaction, for shy students themselves, shyness may predict students’ academic engagement. Firstly, shy students are perceived to have low confidence in their academic abilities, which may reduce their willingness to demonstrate academic achievement and thus affect their participation in demonstrative learning activities [29,30]. Secondly, shy children feel wary and anxious in situations where they believe they will be socially judged [31,32]. In the school setting, academic assessment that focus on academic performance have the potential to stimulate anxiety in shy students [1]. In addition, anxiety levels are predictors of academic engagement [33]. Thus, the academic assessment in the school may influence academic engagement. Although, Hughes and Coplan’s study evidenced that shyness had a significant negative association with academic engagement; the study was conducted with Canadian public-school children aged 9–13 years [6]. However, shyness increases rapidly during adolescence [34]. Whether the association between shyness and academic engagement in adolescence is consistent with that in childhood has not been validated. Therefore, this study attempts to fill this research gap by focusing on college-level adolescents.

When shyness and academic engagement transfer to music education, there is limited research on the association between the two. However, there are rationales to predict the relationship between shyness and music academic engagement. In the context of music learning, music learning emphasizes not only understanding what music is about and also music ‘within’ [35]. High-quality musical experiences and engagement are thought to help music learners ‘within the music’, which is vital to music learning [36]. As academic engagement is defined as an interaction between a student and the educational environment [8], music academic engagement therefore implies an interaction between a student and the educational environment of music. Academic engagement in music educational environment emphasizes collaboration, performance and responsiveness and is highly demanding of social interaction [9]. Collaboration in music education activities may cause shy students to avoid social interactions, which may negatively predict students’ music academic engagement [6]. At the same time, musical performance activities in music education may cause a certain number of negative emotions, such as anxiety, in some of the shy students who lack confidence or have low self-esteem [37]. Besides, Music Performance Anxiety (MPA) is a symptomatology that represents the persistent apprehension and anxiety that compromises the music performance, whether in the form of solo or group performances [38]. It has been shown that MPA influence students who study and engage in music performance [39]. Rodríguez-Mora and Díaz’s research showed a positive correlation between MPA and neuroticism and a negative correlation with extraversion and concluded that a personality prone to neuroticism and introversion seems to have an influence on the MPA [40]. In addition, shyness was predicted by introversion and neuroticism [41]. Hence, shyness might positively influence MPA, which in turn negatively predicts the music academic engagement in educational music performance activities [6,33,42]. Therefore, this study hypothesizes that shyness may negatively predict music academic engagement (Hypothesis 1).

### 2.2. Shyness and Achievement Goals

According to the Models of Personality and Affect regarding the role of psychological constructs in educational contexts [14], including first-level variables—dispositional constructs (e.g., personality traits), second-level variables—motivational processes, third-level variables—educational outcomes (e.g., classroom behavior, etc.), there is an association among them, which is that first-level variables influence the third-level variables with the help of the second-level variables. As we mentioned before, shyness is a type of personality which can be categorized as a first-level variable [1], academic engagement can be categorized as the third-level variable, while shyness had a significant negative association with academic engagement [6]. In addition, we found that shyness is defined as constraint and anxiety when confronted with new objects or perceived social evaluations in social situations and it is thought to result from approach and avoidance motivations in social situations [1,7]. The approach–avoidance motivation is an integral part of achievement motivation, which shows the association between shyness and achievement motivation [43]. Meanwhile, achievement motivation could predict student engagement [44,45]. Following this line of thought, whether achievement motivation is the second-level variable influencing shyness and academic engagement attracted our attention.

Elliot and Church proposed a hierarchical model of approach and avoidance achievement motivation [46]. Within the model, achievement motivation is categorized into different achievement goals. Achievement goals are defined as the purpose of task engagement as a way of explaining an individual’s ability-related effort [46], which could influence individual motivation and individual achievement behavior and is an important component of student motivation and self-regulated learning [47]. Different types of achievement goals unpack the explanations, experiences and actions associated with the individual’s pursuit of achievement [48]. Initially, achievement goal theory was conceptualized in two directions, namely performance goals that focus on demonstrating competence and mastery goals that focus on developing competence and mastery of the task [49]. Furthermore, there is also a categorization based on mastery—performance and approach–avoidance distinctions and divides the achievement goals into four orientations, namely mastery-approach, performance-approach, mastery-avoidance and performance-avoidance [50]. The categorizations discussed above both conceptualize achievement goals by a performance goal versus mastery goal dichotomy. However, Elliot and Harackiewicz categorized performance goal orientation into independent approach and avoidance motivational orientations and three achievement orientations have been proposed, namely mastery goals, performance-approach goals and performance-avoidance goals [51]. The reason for this division is that researchers have experimentally found that only performance goals grounded in the avoidance of failure, which means that only performance goals have an approach-avoidance dichotomy, while mastery goals do not have an avoidance dimension, therefore, mastery-avoidance goals should not as a dimension of achievement goals [51,52].

There is limited research on the relationship between shyness and achievement goals. However, shyness is subgroups of social withdrawal [53]. Meanwhile, in the school setting, social withdrawal is the represent feature of punishment sensitivity, which is one of the basic systems in reinforcement sensitivity theory [54]. Reinforcement sensitivity theory, which includes reward sensitivity and punishment sensitivity, explains the innate tendency of individuals to be sensitive to rewards or punishments in the environment and respond to approach or avoidance behaviors [55]. The research has shown that temperamental sensitivities are associated with achievement goal orientations [56]. Thus, shyness might be associated with achievement goal orientations. Based on the above discussion, the present study further unpacks achievement motivation and attempts to explore the predictive role of shyness on three achievement goals. Mastery goals that focus on developing competence and mastery of the task, performance-approach goals focus on making positive judgments about competence, and performance-avoidance goals avoid making negative judgments about competence [46]. More specifically, mastery goals, which emphasize self-evaluation of one’s abilities, are mostly self-oriented or task-oriented and contribute to intrinsic motivation. Shyness was a negative predictor of intrinsic motivation [57]. Thus, shyness was negatively associated with mastery goals [16]. In addition, shy people are more likely to have low self-esteem [58]. Meanwhile, students with low self-esteem were thought to have lower perceptions of mastery goals [15], which also validates this result. Performance goals, which emphasize leaving the judgment of one’s abilities to others, lead to individuals being more susceptible to extrinsic motivation. Researchers found that shy individuals are prone worry about self-presentation and the judgments of others [31,34,59]. It follows that shy individuals may be vulnerable to extrinsic motivation. Studies have identified that shyness was positively associated with performance-approach goals and performance-avoidance goals [16]. Therefore, shyness is considered to be associated with achievement goals. However, limited research has been conducted on shy students and music achievement goals in music learning. Therefore, based on these findings, the present study made the hypothesis that shyness might associated with achievement goals.

### 2.3. Achievement Goals and Music Academic Engagement

Different achievement goals students hold in academic settings can make qualitative differences in their cognitive, affective and behavioral processes and outcomes [60]. Achievement goals have been found to predict students’ academic engagement. More specifically, it has been found that mastery goals positively predict academic engagement and are negatively associated with behaviors such as withdrawal and avoidance of challenges [17,61]; Performance-approach goals negatively predict academic engagement [61,62]. They are consistently associated with negative behaviors, such as low engagement, higher levels of withdrawal and challenge avoidance [61], and are related to avoidant help-seeking, and self-handicapping [63]; performance-avoidance goals negatively predict academic engagement, as they influence students’ self-control strategies, disruptive behaviors and task disengagement [64]. Although achievement goals have been shown to be related to academic engagement, there is a research gap in the association between achievement goals and music academic engagement in the field of music education. Considering the above discussion, this study hypothesizes that achievement goals might be associated with music academic engagement. As discussed above, shyness could predict achievement goals [16]. In addition, researchers found that achievement goals are considered helpful in understanding students’ engagement and could predict the achievement engagement [65]. Thus, achievement goals may mediate shyness and academic engagement. However, given the discussion above, there is a research gap in this area in the field of music education. Therefore, this study hypothesizes that achievement goals may mediate shyness and music academic engagement (Hypothesis 2).

### 2.4. The Current Study

**Figure 1:**
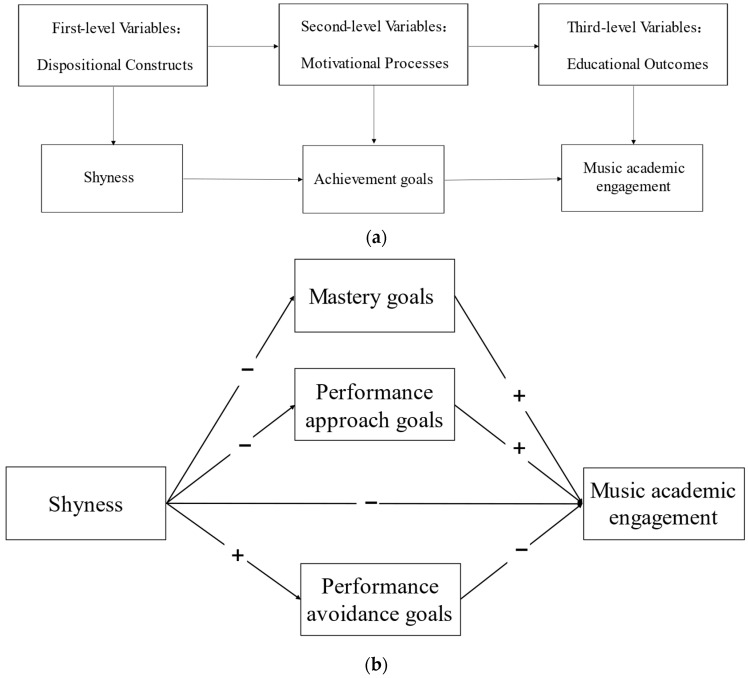
(**a**) The Models of Personality and Affect and hypothesis of the relation among achievement goals, shyness and music academic engagement. (**b**) Hypothesis model of the relation among achievement goals, shyness and music academic engagement.

Based on the above discussion, this study proposed the two hypotheses. Previous research has found that shyness is negatively related to academic engagement [6]. Therefore, when brought into the music learning context, the first hypothesis of this study is that shyness is negatively related to music academic engagement. The second hypothesis in the study comes from two aspects. Firstly, the role of psychological constructs in educational contexts is framed in the Models of Personality and Affect: (1) the first-level variables are dispositional constructs (e.g., personality traits); (2) the second-level variables are motivational processes, and with the help of the second-level variables, the first-level variables have an impact on (3) educational outcomes such as classroom behavior, etc. [14]. In other words, personality traits are fundamental to an individual’s classroom behavior [66,67]. Based on the Models of Personality and Affect, this study included shyness as a personality trait in the first level of variables, engagement in learning as part of educational outcomes and achievement goals as the second level of motivational variables. Secondly, after the above discussion of the existing views and studies. The researcher found that shyness was associated with academic engagement, while achievement goals was associated with both shyness and academic engagement. Therefore, the second hypothesis of this study expects to identify the music achievement goals mediate shyness and music academic engagement (See Figure 1a). The hypothesis model of the relation among music achievement goals, shyness and music academic engagement is shown below (See Figure 1b).

## 3. Methods

### 3.1. Participants

Because public universities are the main type of higher education in China, we selected college students majored in music as the participants in a public university, which means that they can represent the general music college students. We used the cluster random sampling method to select these participants from a public university located in Shanxi Province as this province represents the middle-income regions of China. The department of music in this university has about 600 undergraduates, and 515 participants finally volunteered to complete the questionnaire. So, in current study, 515 college students majoring in music were randomly recruited from a public university in Shanxi province, China in May 2022 through a quantitative survey. The average age of participants was 19.12 with the standard deviation equal to 1.75 (from 15–26 years old). Among them, 198 (38.4%) participants were girls and 317 (61.6%) participants were boys. All participants reported studying music-related majors.

### 3.2. Procedures

First, the research procedures and project were approved by the academic ethics committee of the first author’s school. Second, written informed consent and consent forms were provided to students participating in the study. For 23 students under 18 years of age, we obtained their parents’ permission. They were told that their responses to the questionnaire would be anonymous and confidential, and that the data collected would be used only for academic research. Finally, trained research assistants distributed and collected questionnaires using *wenjuanxing*, an online questionnaire publishing platform (https://www.wjx.cn/, accessed on 10 May 2022). The participants took about 20 min to complete the self-report questionnaire.

### 3.3. Measures

#### 3.3.1. Shyness

The trait of shyness was measured by a shyness scale which was developed by previous researchers [68] and has been recently proved to be suitable for the Chinese context [69]. The scale consists of 9 items (e.g., I feel nervous when speaking to someone in authority). Participants were asked to rate how much they agreed or disagreed with each using a five-point Likert scale (“1 = strongly disagree” and “5 = strongly agree”). After converting the reverse items, the higher scores showed the higher level of shyness. In the current study, the results of Confirmatory Factor Analysis (CFA, χ^2^/df = 90.818/23, CFI = 0.973, TLI = 0.957, RMSEA = 0.076, SRMR = 0.030) indicated the good fit indices, and the Cronbach α value (0.808) for this scale showed a good internal consistency reliability.

#### 3.3.2. Music Achievement Goals

College students’ music achievement goals were assessed by a Chinese adapted scale of the achievement goals questionnaire [46] which has been used in a music context [70]. The scale has 16 items with three aspects including mastery goals (e.g., It is important for me to have a deep and thorough understanding of music theory and music works), performance approach goals (e.g., It is important for me to master more theoretical knowledge and better playing skills than others) and performance avoidance goals (e.g., I often worry that I will study worse than most students in my class.). All items were assessed on a 5-point Likert scale, ranging from 1 (completely disagree) to 5 (completely agree), with higher scores indicating a higher level of trait self-regulation. The CFA results of mastery goals showed the good structural validity (χ^2^/df = 8.615/3, CFI = 0.997, TLI = 0.989, RMSEA = 0.060, SRMR = 0.009) and good internal consistency reliability (Cronbach’s alpha = 0.907). Additionally, the dimension of performance approach goals (χ^2^/df = 22.623/7, CFI = 0.993, TLI = 0.984, RMSEA = 0.066, SRMR = 0.016; Cronbach’s alpha = 0.914) and performance avoidance goals also showed good structural validity and good internal consistency reliability (χ^2^/df = 14.406/5, CFI = 0.992, TLI = 0.985, RMSEA = 0.060, SRMR = 0.018; Cronbach’s α = 0.875) in the present study.

#### 3.3.3. Music Academic Engagement

The music academic engagement questionnaire adopted from previous research [71,72] was used to measure college students’ cognitive engagement (“I’ll check my music homework to make sure it’s correct”) and behavioral (“In music theory and professional courses, I stayed focused”) and emotional (“I enjoy the theoretical knowledge and professional challenges of music”) characteristics, respectively. Participants were asked to rate their music academic engagement on a Likert-type scale ranging from 1 (strongly disagree) to 5 (strongly agree). In the current study, the fit index displayed the good structural validity (χ^2^/df = 401.385/95, CFI = 0.960, TLI = 0.950, RMSEA = 0.079, SRMR = 0.028). In addition, the Cronbach’s α of these 30 items was 0.899, indicating the average scores of these items were used to represent participants’ engagement appropriately.

### 3.4. Data Analyses

To answer the research question, we mainly used the statistic software of SPSS 26.0 with PROCESS macro (http://www.afhayes.com, accessed on 21 December 2018) [73] to conduct the data analyses. First, the descriptive statistics and correlation analysis were performed to acquire the primary descriptive results. Second, linear regression was used to examine the predictive relation between independent variable and dependent variable. Finally, the bias-corrected bootstrapping method was used to check the path coefficient and significance of mediating effects. According to Hayes’s suggestion, if the 95% confidence interval does not contain zero, it indicates a significant predictive effect [73].

The model fit quality was assessed by the following criteria [74]: χ^2^ likelihood ratio tests, the comparative fit index (CFI), the Tucker–Lewis index (TLI), the root mean square error of approximation (RMSEA) and the standardized root mean residual (SRMR). Following the suggestion of Wen et al., the reasonable cut-off value of the acceptable model fitting index was as follows: CFI and TLI no less than 0.90, and RMSEA and SRMR no more than 0.08 [75].

## 4. Results

### 4.1. Preliminary Analyses

Table 1 demonstrates the results of correlations and descriptive analysis for the main research variables. As expected, shyness was positively correlated with performance avoidance goals, but was negatively correlated with mastery goals, and music academic engagement. Additionally, mastery goals were significantly and positively associated with performance approach goals, performance avoidance goals and music academic engagement. Performance approach goals were also positively associated with performance avoidance goals and music academic engagement. The mean value for shyness, mastery goals, performance approach goals, performance avoidance goals, and music academic engagement were 3.03, 4.18, 3.92, 3.78 and 3.73. Due to the total score of these variables are all 5, the average level of shyness among these participants was relatively low but the average level of mastery goals and performance approach goals was relatively high. The SD for main variables wave ranged from 0.469 to 0.831. Among them, the SD of music academic engagement was smallest, while the SD of performance avoidance goals was largest. These results meant that the degree of dispersion (i.e., between-participants differences) of music academic engagement was small but the individual differences of performance avoidance goals was relatively large. The details of age and gender could be shown in Tables S1 and S2.

### 4.2. Testing for the Hypothetical Model

To verify Hypothesis 1, a direct linear regression model of shyness and music academic engagement was built. The results showed that shyness trait positively predicted students’ music academic engagement even after controlling for the effects of gender and age (β = −0.197, *p* < 0.001). So, Hypothesis 1 was supported.

**Figure 2:**
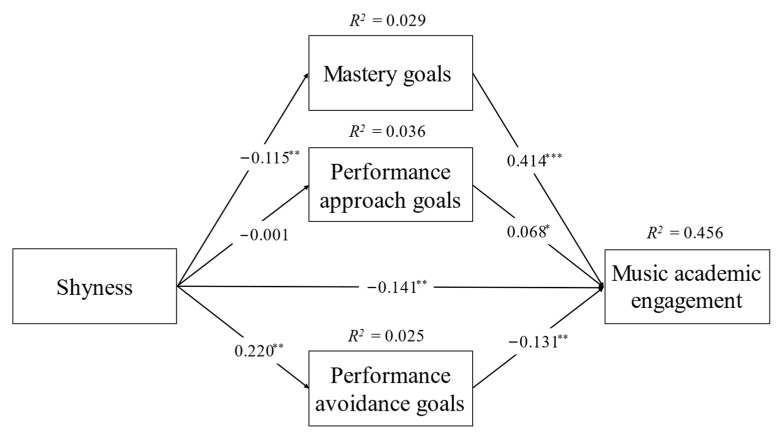
Mediation model of music achievement goals between shyness and music academic engagement. Note: * *p* < 0.05, ** *p* < 0.01, *** *p* < 0.001.

Next, to verify Hypothesis 2, the mediation model with music achievement goals as mediators between shyness and music academic engagement was formulated using the No. 4 Model in PROCESS macro [73]. The results in Table 2 showed that shyness was negatively associated with mastery goals (β = −0.108, *p* < 0.01, 95% CI: [−0.196, −0.021]) and music academic engagement (β = −0.126, *p* < 0.001, 95% CI: [−0.175, −0.078]), and was positively associated with performance avoidance goals (β = 0.175, *p* < 0.01, 95% CI: [0.063, 0.287]), but was not significantly associated with performance approach goals (β = −0.019, *p* > 0.05, 95% CI: [−0.118, 0.079]). Additionally, mastery goals (β = 0.419, *p* < 0.001, 95% CI: [0.361, 0.473]) and performance approach goals (β = −0.068, *p* < 0.01, 95% CI: [0.011, 0.125]) were positively associated with music academic engagement; however, performance avoidance goals were negatively associated with music academic engagement (β = −0.137, *p* < 0.001, 95% CI: [−0.182, −0.093]). The path coefficient and adjusted R-square were also displayed in Figure 2.

Finally, the 95% confidence interval of mastery goals, as a judgment index of mediating effect, ranged from −0.079 to −0.010 (not including zero), and the 95% confidence interval of performance avoidance goals ranged from −0.047 to −0.006 (not including zero), but the 95% confidence interval of performance approach goals ranged from −0.010 to 0.007 (including zero). These findings indicated that mastery goals and performance-avoidance goals (but not performance approach goals) partially mediated the relation between shyness and music academic engagement, partially supporting Hypothesis 2.

## 5. Discussions

### 5.1. Shyness Negatively Predicts Music Academic Engagement

This study aimed to explore the association between shyness and music academic engagement in music learning context. This study concludes that shyness is negatively related to musical academic participation. In other words, the shyer the students, the worse the students’ academic engagement in music learning. Such a conclusion is consistent with the correlation between shyness and non-musical academic engagement [6,76]. It also proves that shyness and academic engagement do not change their correlation when transferred to the context of music learning context. The finding proves the possibility of speculations that shy students are prone to negative cognition and low self-esteem due to lack of and fear of social interaction and generate tension, anxiety and avoidance, which reduce students’ music academic engagement [16]. However, these speculations still need to be further unpacked and evaluated. Furthermore, according to the discussion in the literature review section, the emphasis on experience, performance, participation, and collaboration in the music discipline seems to be more likely than in other disciplines to cause negative emotions in shy students and further impact students’ academic engagement [11,12,13,37]. This study does not provide sufficient evidence of whether and how the characteristics of the music discipline impact differently on shy students compared to other disciplines. Hence, it is also a future research direction for this study.

### 5.2. The Mediating Role of Achievement Goals

The findings indicate that the music mastery goal and the music performance-avoidance goals (excluding the performance approach goal) partially mediated the association between shyness and music academic engagement in music learning. More specifically, firstly, shyness was negatively related to the music mastery goals and shyness was positively related to the music performance-avoidance goals, but not significantly related to the music performance-approach goals. Second, the music mastery goals and the music performance-approach goals were positively related to music academic engagement, while the music performance-avoidance goal was negatively related to music academic engagement.

The findings that shyness and music mastery goals are negatively related are consistent with existing research [16]. Transferring to music learning did not change the relationship between the two. When students value mastery goals, they are usually able to enjoy the learning itself and have a higher sense of self-efficacy, so mastery goals usually promote more active classroom participation [77]. However, for shy students, lack of confidence and the tendency to have biased negative perceptions make shy students prone to self-doubt [59]. Thus, they are less likely to make mastery goals their achievement goals [15,16]. This study found that shyness was positively associated with music performance-avoidance goals, but not significantly associated with music performance-approach goals. Exploring whether musical disciplines, such as performance, collaboration and other components of music learning, are related to the changes in the relationship between shyness and music performance-approach goals is also one of the future directions of this study.

The finding that music mastery goals and music performance-approach goals were positively related to music academic engagement and that music performance-avoidance goals were negatively related to music academic engagement are consistent with existing research [61]. Whether intrinsically or extrinsically motivated, shy students are likely to adopt music mastery goals and music performance-approach goals in order to avoid failure in music learning (e.g., performance) and thus engage in more music academic engagement [37]. Conversely, for shy students, lack of confidence and proneness to negative perceptions may prompt students to directly choose the music performance-avoidance goals, resulting in lower music academic engagement [59]. However, these inferences need to be further examined.

## 6. Conclusions

This research revealed the following conclusions: (1) shyness is negatively associated with musical academic engagement; (2) the music mastery goal and the music performance-avoidance goals (excluding the performance approach goal) partially mediated the association between shyness and music academic engagement in music learning. These findings have implications for the research and practice of music academic engagement of shyness. There are both theoretical and practical implications for this study. For the theoretical implications, this study transferred shyness and academic engagement to a music learning context and found a negative association between shyness and music academic engagement in a music learning context. In the meantime, this study explored the differentiated mediation role of multi-dimensional music achievement goals in the association of shyness and music academic engagement. This study fills a gap in the current research on shyness and academic engagement in music learning contexts. At the same time, as the study was conducted with Chinese university students, the proposed model also provides a reference for studying the academic engagement of shyness in Chinese contexts. For the practical implication, this study offers a reference for the implementation of music education in the Chinese context. For music teachers, the findings of this study may help music teachers to detect and notice the motivational goal orientations of shy students and provide appropriate music learning activities and enhance shy students’ academic engagement in music learning. Firstly, this study verifies the association between shyness and music academic engagement. To improve shy students’ music academic engagement, music teachers should perceive students’ shyness and try to use teaching strategies to relieve students’ shyness. For example, college music teachers could help ease their students’ shyness and enhance their academic engagement by encouraging students to pay full attention in music learning and act with confidence [78], trying not to be judgmental, setting reasonable goals for shy students, avoiding pushing students and avoiding use the shy label to students [79]. In addition, studies have shown that shyness was significantly and negatively related to close teacher–child relationships and significantly and positively associated with dependent teacher–child relationships. Therefore, music teachers could alleviate students’ shyness by maintaining a close teacher-student relationship [80]. Meanwhile, a study on shyness and college students’ physical education found that group counselling can influence multiple dimensions of student shyness and reduce the degree of shyness. College music teachers could also apply such an approach to ease the degree of students’ shyness [81,82]. At the same time, research has shown that there is an association between teachers’ teaching and students’ academic engagement. For example, there is a significant but moderate relationship between teachers’ teaching styles and students’ academic engagement [83]. Furthermore, teachers’ instructional design [84], academic support [85], teacher need-supporting practices (autonomy support, structure, and involvement) [86] and teaching learning strategies [87] contribute to students’ academic engagement. In addition, research on academic engagement helps to understand the quality of students’ learning experiences and helps teachers to determine and provide instructional resources and course content [23]. Despite the lack of relevant studies on music education, the study in the context of English education has suggested that English as a Foreign Language learners with a high degree of shyness may keep silent in the course, avoid participating in cooperative tasks, and tend to use some avoidance strategies in the face of pressure. Language teachers should adopt various skills and appropriate tasks to help these students overcome their negative emotional characteristics and promote students’ academic participation [88]. Hence, based on the findings of this study, in order to promote shy students’ music academic engagement, the teachers should not only relieve the degree of shyness for students but also use the teaching strategy or guiding shy students to use mastery goals and performances-approach goals and avoid use performance-avoidance goals. Research has shown that a master-oriented classroom environment which is characterized by providing students with motivating tasks, and autonomy support could encourage students to adopt a mastery goal orientation [89]. In addition, teachers could set concrete performance goals for their students based on their ability levels and guide them to plan appropriate strategies to encourage them to use performance approach goals orientation and achieve the goals [90]. Besides, teachers should be vigilant and avoid shy students using performance-approach goals, including the use of self-handicapping strategies, avoidance of novelty and challenges, and reluctance to cooperate with peers [91].

Although this study has some theoretical and practical implications, it also has limitations. Firstly, the data for this study were derived from a self-report method. Although the self-report method is considered a classic research paradigm in the field of personality and motivation research [92], there are differences in subjects’ introspective abilities. In the future, more experimental task paradigms would be applied to avoid subjective bias caused by differences in subjects’ introspective abilities; secondly, the sample for this study was collected from one university in China, and the sample size may limit the generalization of the study findings. Future research may consider expanding the subject population of the study to enhance the external validity of the study and the generalizability of the findings; finally, the framework of cognitive, behavioral and affective was used to examine learning engagement in this paper. Other researchers have studied academic engagement from the framework of vigor, dedication and absorption [93]. For this study, whether the current relations of variables are valid under this theoretical framework (i.e., vigor, dedication and absorption) is a promising direction for future research.

## Figures and Tables

**Table 1:** Means, standard deviations, and correlations statistics.

Variables	M	SD	Shyness	Mastery Goals	Performance Approach Goals	Performance Avoidance Goals	Music Academic Engagement	Gender	Age
Shyness	3.03	0.647							
Mastery goals	4.18	0.648	−0.114 **						
Performance approach goals	3.92	0.732	−0.029	0.501 ***					
Performance avoidance goals	3.78	0.831	0.130 **	0.222 ***	0.551 ***				
Music academic engagement	3.73	0.469	−0.272 ***	0.594 ***	0.267 ***	−0.072			
Gender			0.063	−0.126 **	−0.177 ***	−0.035	−0.043		
Age	19.12	1.749	0.019	−0.009	−0.053	−0.073	−0.117 **	−0.069	

Note: ** *p* < 0.01, *** *p* < 0.001.

**Table 2:** Mediation model of music achievement goals between shyness and music academic engagement.

Predictors	Mastery Goals			Performance Approach Goals			Performance Avoidance Goals			Music Academic Engagement		
	β	SE	95% CI	β	SE	95% CI	β	SE	95% CI	β	SE	95% CI
Shyness	−0.108 **	0.045	[−0.196, −0.021]	−0.019	0.050	[−0.118, 0.079]	0.175 **	0.057	[0.063, 0.287]	−0.126 ***	0.025	[−0.175, −0.078]
Mastery goals										0.419 ***	0.028	[0.361, 0.473]
Performance approach goals										0.068 *	0.029	[0.011, 0.125]
Performance avoidance goals										−0.137 ***	0.023	[−0.182, −0.093]
Gender	−0.160 **	0.058	[−0.275, −0.046]	−0.272 ***	0.066	[−0.401, −0.143]	−0.084	0.075	[−0.231, 0.063]	0.041	0.033	[−0.023, 0.105]
Age	−0.006	0.016	[−0.038, 0.026]	0.028	0.018	[−0.063, 0.008]	−0.037 *	0.021	[−0.078, 0.003]	−0.032	0.009	[−0.049, −0.014]
*R* ^2^	0.027			0.036			0.025			0.449		
*F*	4.805			6.380			6.344			68.856		

Note: * *p* < 0.05, ** *p* < 0.01, *** *p* < 0.001.

## Data Availability

Data will be made available on request.

## Supplementary Materials

The following supporting information can be downloaded at: https://www.mdpi.com/article/10.3390/ijerph20010824/s1, Table S1: The descriptive statistics of age; Table S2: The descriptive statistics of gender.
