# Supplementary material for: Relation between Shyness and Music Academic Engagement: The Mediation of Achievement Goals—A Cross-Sectional Survey Study
Source: Int J Environ Res Public Health. 2023 Jan 1;20(1):824. doi: 10.3390/ijerph20010824 (PMC9819276; doi:10.3390/ijerph20010824)
Supplement: Supplementary file 1 [file ijerph-20-00824-s001.zip › ijerph-2112834-supplementary.pdf]

Supplementary Materials

Table S1. The descriptive statistics of age.

**Age**	**N**	**%**
15	1	.2
17	22	4.3
18	189	36.7
19	146	28.3
20	78	15.1
21	43	8.3
22	10	1.9
23	13	2.5
24	6	1.2
25	6	1.2
26	1	.2
Total	515	100.0

Table S2. The descriptive statistics of gender.

Gender	N	%
Boys	198	38.4
Girls	317	61.6
Total	515	100.0
